# Estimation of Nitrogen Content in Alfalfa Plants Based on Multi-Source Feature Fusion

**DOI:** 10.3390/plants15050752

**Published:** 2026-02-28

**Authors:** Jiapeng Zhu, Haohao Dang, Demin Fu, Guangping Qi, Yanxia Kang, Yanlin Ma, Siqin Zhang, Chungang Jing, Bojie Xie, Yuanbo Jiang, Jinxi Chen, Boda Li, Jun Yu

**Affiliations:** 1 College of Water Conservancy and Hydrpower Engineering, Gansu Agricultural University, Lanzhou 730070, China; 1073324020383@st.gsau.edu.cn (J.Z.); 1073325121001@st.gsau.edu.cn (D.F.); mayl@gsau.edu.cn (Y.M.); jiebj@gsau.edu.cn (B.X.); 1073324120804@st.gsau.edu.cn (J.C.);; 2 Qingyang Hydrological and Water Resources Survey Center, Qingyang 745000, China; yanxiakang@126.com

**Keywords:** alfalfa, plant nitrogen content, feature fusion, UAV multispectral, machine learning

## Abstract

Plant nitrogen content (PNC) is a core physiological parameter characterizing crop nitrogen nutrition status. Its precise and dynamic monitoring is crucial for crop growth diagnosis, optimizing nitrogen fertilizer management, enhancing fertilizer use efficiency, and reducing agricultural nonpoint source pollution. This study utilized multispectral imagery from unmanned aerial vehicles (UAVs) to extract vegetation indices (VIs) and texture feature values (TFVs) during critical growth stages of alfalfa. By combining TFVs to construct texture indices (TIs), variables exhibiting extremely significant correlations with alfalfa PNC (*p* < 0.001) were identified. We used VIs, TIs, and their combined features as model inputs. The performance of four machine learning models—random forest regression (RFR), Support Vector Regression (SVR), Backpropagation Neural Network (BPNN), and gradient boosting (XG-Boost)—was comprehensively assessed for estimating alfalfa PNC. Our results indicate the following: (1) The correlation coefficients |r| between VIs and alfalfa PNC ranged from 0.56 to 0.68; TIs constructed from TFVs significantly enhanced PNC correlation compared to raw texture values, with |r| exceeding 0.6. (2) Integrating VIs and TIs substantially improved the accuracy of PNC estimation models across growth stages. Compared to using VIs or TIs alone, the validation set *R*^2^ increased by 5.4–19.7%, 1.7–16.4%, and 5.2–17.2% for the branching, budding, and initial flowering stages, respectively. (3) The XG-Boost model demonstrated optimal performance across all growth stages and input variables. Particularly during the budding stage, the VIs + TIs model achieved the highest fitting accuracy: training set *R*^2^ = 0.81, RMSE = 0.15%; validation set *R*^2^ = 0.80, RMSE = 0.12%. In summary, integrating multispectral vegetation indices and texture indices effectively enhances the accuracy of PNC estimation in alfalfa, providing scientific support for precision field management and fertilization decisions in alfalfa cultivation.

## 1. Introduction

Nitrogen is a core element constituting vital substances such as crop proteins, chlorophyll, and nucleic acids, with its supply level directly impacting crop yield formation and nutritional quality [1]. Accurate nitrogen management is vital for safeguarding food security and supporting sustainable agricultural growth. However, excessive nitrogen fertilizer application leads to soil and water pollution, while insufficient nitrogen causes physiological disorders, growth limitations, and reduced stress tolerance in crops, ultimately affecting yield and quality [2,3]. Therefore, rapid and accurate monitoring of crop nitrogen content is vital for rational nitrogen fertilizer application, crop yield estimation, and adjustment of irrigation and fertilization strategies. Traditional crop nitrogen content monitoring relies on manual sampling and chemical analysis. This method not only damages plant integrity but also suffers from time-consuming analysis processes, resulting in significant delays. Furthermore, its labor-intensive and costly nature limits rapid field diagnosis of crop nutritional status [4]. Recently, the rapid progress in remote sensing technology has created new opportunities for monitoring crop growth parameters. Among these, unmanned aerial vehicle (UAV) remote sensing systems have become an essential tool for crop phenotyping and nutritional assessment. Their unique advantages—including operational flexibility, cost-effectiveness, high spatial resolution, and on-demand data acquisition—provide reliable technical support for smart agriculture practices [5,6,7].

Plant nitrogen concentration (PNC) serves as a crucial indicator for assessing crop nitrogen nutrition status [8]. Until now, numerous researchers have employed specific spectral band combinations to construct vegetation indices (VIs) for estimating crop nitrogen content. Wei et al. [9] and Liu et al. [10] achieved precise nitrogen estimation for summer maize and winter wheat by optimizing vegetation indices, analyzing their correlation with leaf nitrogen concentration (LNC), and utilizing optimal spectral variables during critical growth stages. LEE et al. [11] further explored the application of different VIs in estimating maize canopy leaf nitrogen content, finding that VIs derived from the red-edge and near-infrared bands yielded the best estimation performance, significantly improving model accuracy. SHENDRYK et al. [12] extracted multiple features from VI images generated using drone multispectral imagery to achieve inversion of sugarcane LNC; Xu et al. [13] employed polarization remote sensing technology, discovering a strong correlation between polarization spectra at specific angles and rice nitrogen content. By extracting characteristic bands using continuous projection and constructing polarization vegetation indices, they significantly improved the accuracy of rice canopy LNC inversion. The aforementioned studies primarily relied on spectral information. While spectral data can characterize vegetation physiological status, type, and density, they struggle to precisely distinguish vegetation types or ecological environments under similar spectral conditions. During the later growth stages with complex canopy structures, using single spectral variables for inversion often leads to spectral “saturation” and is susceptible to interference from lighting conditions and atmospheric environments [14,15]. Texture information, on the other hand, offers valuable insights into the spatial structure and morphology of vegetation, acting as a useful complement to remote sensing imagery for differentiating vegetation types and growth environments [16]. KHOSRAVI et al. [17] found that texture information offers crucial details about vegetation spatial structure and morphology, exhibits strong resistance to image noise, and significantly improves the accuracy of crop nutrient estimation when applied. Combining spectral and textural data enhances the ability to distinguish between data and boosts the performance of regression models [18]. Jia et al. [19] and Guo et al. [20] demonstrated that combining vegetation indices with textural features further enhances the accuracy of wheat plant nitrogen content estimation. Zhang et al. [21] developed STFIs to fuse spectral and texture features, establishing a rice LNC estimation model with an *R*^2^ of 0.87. Fan et al. [22] found that texture indices constructed from multiple texture features strengthened the correlation between texture characteristics and potato PNC, thereby improving the accuracy of potato nitrogen nutrition monitoring. Compared to single-source imagery, integrating spectral and textural information enhances the discernibility of original spatial data [23], overcomes the “saturation” issue in canopy vegetation indices, and improves the reliability of crop nitrogen estimation models. However, existing research has primarily focused on major food crops such as rice and wheat, with relatively limited studies on remote sensing monitoring of nitrogen in alfalfa. Most current studies rely on single spectral information to construct estimation models, with insufficient utilization of texture features. They often directly employ raw texture feature values without systematic optimization or index construction. The texture index constructed through mathematical combinations effectively integrates multi-band texture information, significantly enhancing its correlation with plant nitrogen content. This approach reduces feature dimension and noise interference while strengthening the model’s ability to capture canopy spatial structure information, thereby providing more robust feature inputs for alfalfa nitrogen monitoring. Furthermore, there remains a lack of in-depth exploration on how to integrate spectral and texture features and systematically evaluate their ability to estimate plant nitrogen content across different growth stages of alfalfa. Therefore, this study systematically evaluates the response patterns of multi-source feature fusion at different growth stages by constructing texture index-optimized feature expression, while comprehensively assessing the estimation performance of multiple models. Furthermore, extending this approach to alfalfa effectively addresses the limitations of existing research in nitrogen remote sensing monitoring for forage crops.

Alfalfa (*Medicago sativa* L.), as the most widely cultivated high-quality perennial leguminous forage globally, serves not only as a crucial feed source for sustainable livestock development but also plays an irreplaceable role in soil improvement and fertility enhancement [24]. Therefore, rapid and precise monitoring of alfalfa PNC not only facilitates variable-rate fertilization and enhances nitrogen fertilizer utilization efficiency but also reduces the potential for nonpoint source pollution. This provides a crucial safeguard for achieving high yields and quality production in alfalfa. This study estimates alfalfa PNC as the total nitrogen content of the stem–leaf mixture, which comprehensively reflects the crop’s overall nitrogen nutrition status. For alfalfa harvested as an above-ground whole plant, it serves as a more direct key agronomic indicator for guiding fertilization [8]. Although previous studies have primarily focused on LNC [9,10,11,12,13], PNC is highly correlated with LNC and incorporates nitrogen storage information from the stems. This makes PNC particularly significant for evaluating forage quality and overall nitrogen uptake. This study constructs texture indices by combining texture feature values. It systematically compares the performance of vegetation indices, texture indices, and their fusion within different machine learning models, aiming to: (1) optimize PNC estimation accuracy by reducing data dimensionality through texture feature values (TFVs) and constructed texture indices (TIs); (2) assess the accuracy of alfalfa PNC models using vegetation indices, texture indices, and feature fusion; and (3) use four machine learning models—random forest regression (RFR), Support Vector Regression (SVR), Backpropagation Neural Network (BPNN), and XG-Boost—to estimate alfalfa PNC, aiming to identify the best-performing model. This provides a theoretical basis for precise nitrogen monitoring and intelligent fertilization in alfalfa cultivation. The innovation of this study lies in proposing a multi-source feature fusion and optimization method for alfalfa PNC remote sensing estimation. It overcomes the limitations of directly using raw TFVs by constructing and screening TIs through a mathematical combination system, significantly enhancing feature representation capabilities. The study integrates VIs with TIs and systematically compares the response mechanisms of different machine learning models to these integrated features. Furthermore, this study is the first to demonstrate the stable advantages of feature fusion over single data sources in alfalfa PNC monitoring. It confirms the XG-Boost model as the optimal performer for processing such fused features and successfully applies this technical framework to alfalfa, a vital forage crop. This work lays the foundation for precise nitrogen diagnosis in complex canopy crops.

## 2. Results

### 2.1. PNC Statistical Analysis

**Figure 1:**
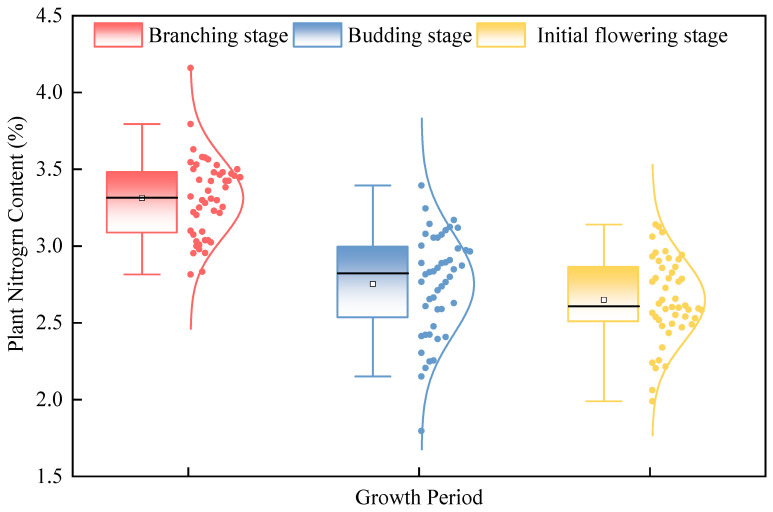
Statistical results of PNC at different growth stages.

Statistical analysis (Table 1 and Figure 1) revealed that the PNC values across the three growth stages of alfalfa followed a normal distribution. Both the standard deviation (0.26–0.33) and coefficient of variation (7.90–12.04%) were relatively low, indicating uniform distribution and low dispersion of PNC values. The average PNC of alfalfa exhibited a decreasing trend with advancing growth stages, with values of 3.31%, 2.75%, and 2.64% at the branching stage, budding stage, and initial flowering stage, respectively.

### 2.2. Variable Filtering

This study employed Pearson correlation analysis to investigate the relationships between vegetation indices, texture feature values, texture indices, and PNC. For each growth stage of alfalfa, vegetation indices with a correlation coefficient |r| > 0.5 and ranking among the top three in correlation strength were selected. Additionally, for each of the three texture index categories, one texture index with |r| > 0.5 and the highest correlation coefficient was chosen.

#### 2.2.1. Correlation Between Vegetation Index (VI) and PNC

**Figure 2:**
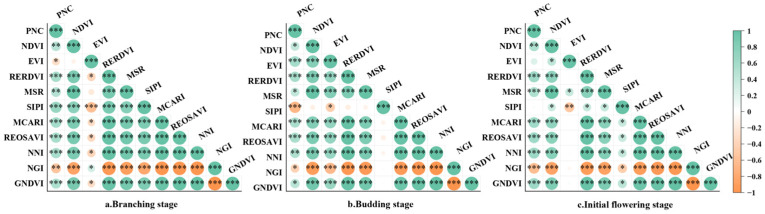
Correlation between PNC and vegetation index. In the figure, * indicates *p* < 0.05, ** indicates *p* < 0.01, *** indicates *p* < 0.001. When the p-value is less than 0.05, the result is statistically significant; when less than 0.01, it is highly statistically significant; and when less than 0.001, it indicates an extremely significant statistical difference. All meet the modeling requirements.

Pearson correlation analysis between vegetation indices and alfalfa PNC (Figure 2) revealed that during the branching stage, PNC showed highly significant correlations with SIPI, MCARI, and REOSAVI, with correlation coefficients of 0.67, 0.59, and 0.57, respectively. At the budding stage, PNC showed highly significant correlations with SIPI, EVI, and RERDVI, with correlation coefficients of −0.68, 0.59, and 0.56, respectively; at the initial flowering stage, PNC showed highly significant correlations with NNI, RERDVI, and GNDVI, with correlation coefficients of 0.63, 0.62, and 0.62, respectively.

#### 2.2.2. Correlation Between Texture Feature Values (TFVs) and PNC

**Figure 3:**
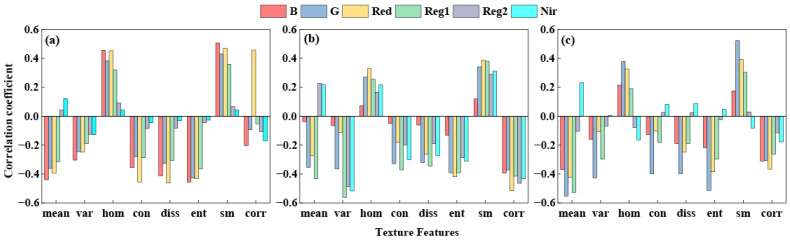
The correlation coefficient between texture features and PNC. (**a**) Branching stage; (**b**) budding stage; (**c**) initial flowering stage.

We performed correlation analysis between single texture features extracted from alfalfa at different growth stages using GLCM and PNC. Results are shown in Figure 3a–c. The correlation between single texture features and PNC is relatively low, with only B_sm at the branching stage, RE1_var, NIR_var, and R_corr at the budding stage, and at the initial flowering stage, G_mean, RE1_mean, G_ent, and G_sm. More than half of the chosen texture features exhibited weak correlations with PNC, indicating that monitoring alfalfa PNC using single texture features is challenging.

#### 2.2.3. Correlation Between Texture Indices (TIs) and PNC

**Figure 4:**
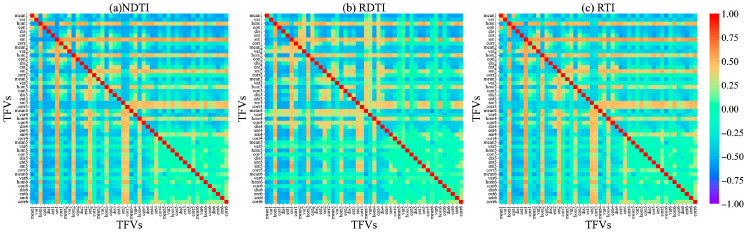
Correlation coefficient between texture index and PNC during the branching stage. In the figure, each point corresponds to the correlation coefficient between the texture index calculated from the x and y coordinates and alfalfa PNC. The numbers 1–6 represent different spectral bands: 1 for the blue band, 2 for the green band, 3 for the red band, 4 for the red-edge 1 band, 5 for the red-edge 2 band, and 6 for the NIR band.

**Figure 5:**
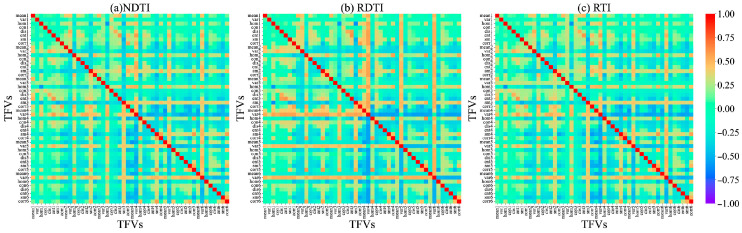
Correlation coefficient between texture index and PNC during the budding stage. In the figure, each point corresponds to the correlation coefficient between the texture index calculated from the x and y coordinates and alfalfa PNC. The numbers 1–6 represent different spectral bands: 1 for the blue band, 2 for the green band, 3 for the red band, 4 for the red-edge 1 band, 5 for the red-edge 2 band, and 6 for the NIR band.

**Figure 6:**
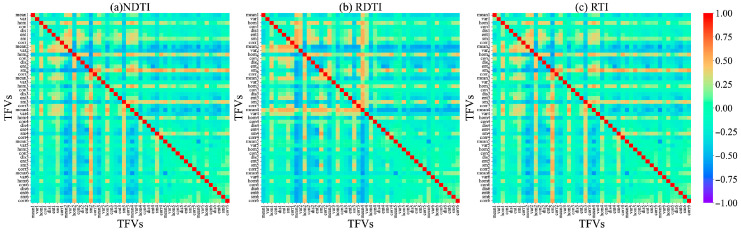
Correlation coefficient between texture index and PNC at the initial flowering stage. In the figure, each point corresponds to the correlation coefficient between the texture index calculated from the x and y coordinates and alfalfa PNC. The numbers 1–6 represent different spectral bands: 1 for the blue band, 2 for the green band, 3 for the red band, 4 for the red-edge 1 band, 5 for the red-edge 2 band, and 6 for the NIR band.

Because of the weak correlation between individual texture features and PNC, this study used texture indices (TIs), which integrate various texture features from different spectral bands, such as the NDTI, RDTI, and RTI. During the branching, budding, and initial flowering stages, the average |r| values for all TFVs were 0.26, 0.30, and 0.23, respectively. In contrast, the average |r| values for the selected optimal TIs (NDTI, RDTI, and RTI) reached 0.62, 0.61, and 0.62, respectively, representing relative improvements ranging from 103.3% to 169.9%. Furthermore, the construction of these optimal TIs integrated texture features across multiple spectral bands (e.g., nir, red-edge, and visible light), indicating that their enhancement effects exhibit cross-band consistency and all reached highly significant levels (*p* < 0.001). At different growth stages of alfalfa, the TIs with the highest correlations with PNC during the branching stage were the NDTI (NIR_mean, B_mean), RDTI (B_sm, NIR_hom), and RTI (NIR_mean, B_mean), with |r| values of 0.62, 0.61, and 0.63, respectively (Figure 4). At the budding stage, they were the NDTI (B_ent, R_ent), RDTI (R_sm, B_hom), and RTI (RE2_corr, RE1_sm), with |r| values of 0.60, 0.62, and 0.61, respectively (Figure 5); at the initial flowering stage, the corresponding indices were the NDTI (G_sm, RE1_hom), RDTI (NIR_mean, RE1_mean), and RTI (G_sm and RE1_hom), with |r| values of 0.62, 0.61, and 0.62, respectively (Figure 6).

### 2.3. Comprehensive Evaluation of Models

**Figure 7:**
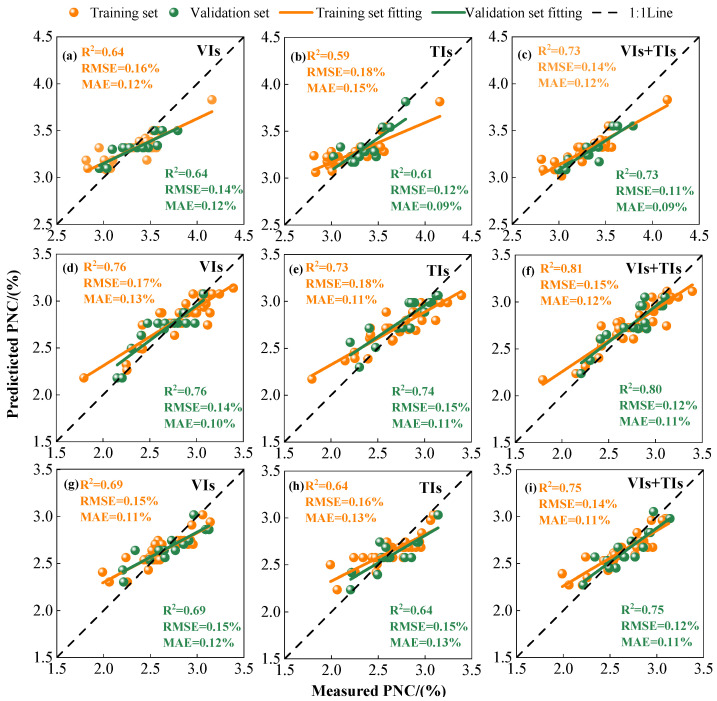
Actual and estimated values of the best PNC prediction model (XG-Boost). In the figure, (**a**–**c**) branching stage, (**d**–**f**) budding stage, (**g**–**i**) initial flowering stage.

This study compared the prediction accuracy of four machine learning models—random forest regression (RFR), Support Vector Regression (SVR), BP neural network, and XG-Boost—on plant nitrogen content (PNC) across different growth stages and various input variables. The results indicate (Table 2) that during the branching stage, when using VIs as input variables, the XG-Boost model achieved validation set *R*^2^, RMSE, and MAE values of 0.64, 0.14%, and 0.12%, respectively. Its *R*^2^ outperformed RFR (0.58), SVR (0.56), and BPNN (0.53). When using TIs as input features, the accuracy of each model decreased slightly, but XG-Boost still maintained the highest accuracy (validation set *R*^2^ = 0.61). When integrating VIs and TIs as input variables, all models demonstrated significantly improved accuracy. Among them, XG-Boost showed the most pronounced enhancement, with training set *R*^2^ increasing by 14.1% compared to VIs alone and by 23.7% compared to TIs alone; validation set *R*^2^ improved by 14.1% over VIs and by 19.7% over TIs (Figure 7a–c).

During the budding stage, XG-Boost demonstrated optimal performance across all three input conditions. When using VIs as input variables, XG-Boost achieved a validation set *R*^2^ of 0.76; when using TIs, it reached 0.74; and when fusing VIs + TIs, it further improved to 0.80, with RMSE and MAE decreasing to 0.12% and 0.11%, respectively. The training set *R*^2^ improved by 6.6% and 11% compared to using VIs or TIs alone, respectively. The validation set *R*^2^ increased by 5.3% and 8.1%, respectively (Figure 7d–f). RFR achieved an *R*^2^ value of 0.71 on the validation set under feature fusion. The estimation accuracy of SVR and BPNN showed only slight improvement, with *R*^2^ values for the validation set remaining below 0.6 for both models.

The trend during the initial flowering stage largely aligns with the previous two growth stages. XG-Boost maintained the highest accuracy across all three input feature sets: VIs, TIs, and VIs + TIs. After feature fusion, the validation set *R*^2^ reached 0.75, with training and validation set *R*^2^ values increasing by 8.7% and 17%, respectively, compared to using VIs or TIs alone (Figure 7g–i). RFR achieved a validated *R*^2^ of 0.63 after feature fusion. Both SVR and BPNN showed only minor improvements in accuracy, with validation set *R*^2^ values below 0.61. In summary, the XG-Boost model demonstrated optimal performance across different growth stages and input variables. When integrating VIs + TIs features, the XG-Boost model achieved an average test set *R*^2^ of 0.76 across three growth stages, significantly outperforming RFR (0.70), SVR (0.60), and BPNN (0.58). Particularly during the budding stage, it demonstrated strong generalization capabilities, with the XG-Boost model achieving validation set *R*^2^, RMSE, and MAE of 0.80, 0.12%, and 0.11%, respectively.

**Figure 8:**
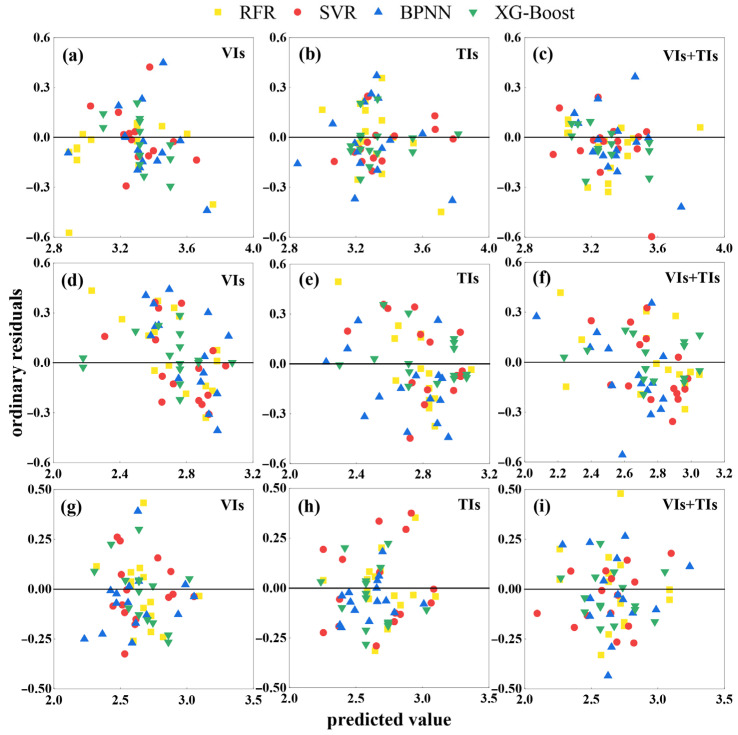
Model residual plot. In the figure, (**a**–**c**) branching stage, (**d**–**f**) budding stage, (**g**–**i**) initial flowering stage.

Residual analysis confirmed the performance of RFR, SVR, BPNN, and XG-Boost models using VIs, TIs, and VIs + TIs as input variables (Figure 8). Models using VIs or TIs as inputs exhibited scattered residual distributions. After integrating VIs + TIs, XG-Boost demonstrated the closest median residual to zero, the smallest interquartile range, and the fewest outliers across all three growth stages. While other models showed improved error distributions compared to single-feature models, their residual dispersion remained significantly higher than XG-Boost. Furthermore, residuals exhibited random distribution within the prediction range, indicating that model errors did not exhibit systematic variation with prediction magnitude, supporting the validity of the model’s design. Simultaneously, residuals fluctuated randomly around the zero line without trend-based clustering, ruling out the possibility of systematic errors in the model.

### 2.4. Spatial Distribution of Nitrogen Content in Plants

**Figure 9:**
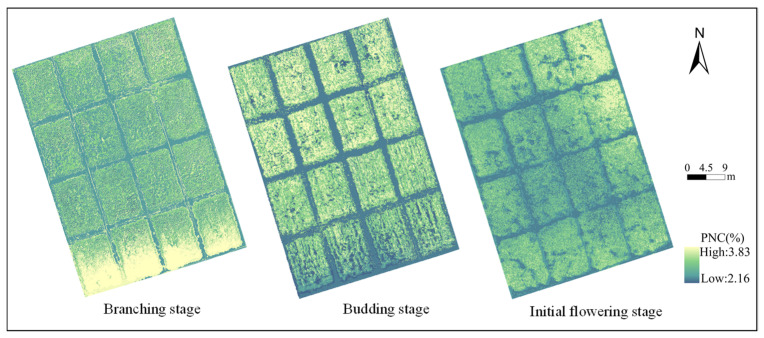
Spatial distribution map of nitrogen content in plants.

This study achieved the highest estimation accuracy using an XG-Boost model with integrated VIs + TIs as input features. Based on this approach, PNC was inverted for three growth stages of alfalfa, ultimately yielding the spatial distribution of PNC across each growth stage (Figure 9). Results indicate a gradual decline in PNC throughout the entire growth period, consistent with measured values. PNC ranged from 2.93% to 3.83% during the branching stage, from 2.17% to 3.13% during the budding stage, and from 2.16% to 3.11% during the initial flowering stage.

## 3. Discussion

### 3.1. Correlation Between Characteristic Variables and Alfalfa PNC

Vegetation indices, texture characteristics, and texture indices exhibit differential responses to alfalfa PNC across different growth stages. The selected vegetation indices exhibit varying sensitivities across different growth stages. During the branching stage, PNC exhibits higher sensitivity to red-edged indices such as the MCARI and REOSAVI. This is closely related to the lower canopy coverage during the early growth stages of crops and the significant influence of soil background. Previous studies on rice have also identified similar trends, consistent with the findings of this research [25]. The red-edged band represents the transition zone where chlorophyll absorption shifts toward leaf scattering, exhibiting extreme sensitivity to variations in chlorophyll concentration. Nitrogen serves as one of the key raw materials for chlorophyll synthesis [26]. The MCARI incorporates red-edged bands to mitigate soil background effects [27], while the REOSAVI further integrates soil correction factors, enabling more effective capture of nitrogen-driven chlorophyll signals before canopy closure [28]; upon entering the budding stage, the Red-Edge Index (RERDVI) and EVI exhibit strong correlations. The EVI corrects atmospheric effects by incorporating the blue light band and exhibits a superior linear response in areas with high biomass, making it suitable for mid-growth stages with complex canopy structures [29]. During the phase when canopy structure becomes more complex and biomass accumulates, these indices are better at distinguishing between vegetation cover and background noise. During the initial flowering stage, correlations for indices like the NNI and GNDVI increase.

This could be due to the nitrogen dilution effect caused by the increase in plant biomass during alfalfa development, which leads to changes in the canopy spectral characteristics. This study also found that single texture features (TFVs) generally showed low correlations with alfalfa PNC (|r| < 0.5) (Figure 3). Shu et al. [30] and Wang et al. [31] found through correlation analysis that the correlation coefficients between TFVs and PNC in rice and winter wheat were generally below 0.5, consistent with the results of this study. While texture features reflect canopy structure and leaf morphology, indirectly characterizing nitrogen uptake and utilization, their extraction is influenced by image resolution, scale transformation, and viewpoint variations. This results in the extraction of numerous texture features, making it difficult to distinguish between valid information and noise [32]. TIs constructed through TFV combinations (NDTI, RTI, and RDTI) significantly enhance correlation with PNC. Post-screening TIs exhibit correlation coefficients |r| > 0.6 (Figure 4, Figure 5 and Figure 6). Zheng et al. [33] significantly improved the correlation with rice PNC by constructing a texture index, consistent with the findings of this study. This indicates that mathematically combined texture indices amplify differences in texture features across different bands, thereby enhancing structural information relevant to PNC.

### 3.2. Complementary Mechanism Integrating Vegetation and Texture Indices

Estimation models for alfalfa PNC based solely on VIs or TIs generally exhibit lower accuracy than models integrating both spectral and texture indices (VIs + TIs), indicating the significant limitations of relying on a single data source for PNC estimation. Possible reasons include the “saturation effect” that VIs exhibit under complex canopy structures or high leaf area indices, which weakens their response to plant nitrogen content [14]. Additionally, factors such as soil background reflectance, atmospheric conditions, and leaf water status can disrupt spectral signal stability. Particularly during early growth stages with high bare soil coverage, soil noise significantly reduces PNC sensitivity to vegetation indices [30]. Furthermore, nitrogen uptake, assimilation, and redistribution processes undergo dynamic changes across crop growth stages, and a single vegetation index struggles to comprehensively capture these spectral response variations driven by nitrogen physiological transport [34]. The construction of TIs relies on feature selection and combination optimization, and is influenced by factors such as image resolution, window size, and canopy geometry [31]. Consequently, PNC estimation models using texture indices as input variables consistently exhibit lower accuracy than those integrating VIs and TIs.

By combining VIs and TIs, the limitations of using a single data source to estimate nitrogen content in alfalfa plants are effectively addressed. In this study, integrating VIs + TIs as input parameters for RFR, SVR, BPNN, and XG-Boost models improved estimation accuracy across all models. The optimal model (XG-Boost) demonstrated higher accuracy in estimating PNC across the three reproductive periods, with *R*^2^ values of 0.73, 0.80, and 0.75, respectively (Table 2). This indicates that feature fusion significantly enhances the model’s ability to characterize alfalfa PNC. This improvement stems from the complementary nature of vegetation indices (VIs) and texture indices (TIs) in resisting interference. VIs primarily reflect one-dimensional spectral information of the canopy, while TIs provide two-dimensional spatial texture information [31]. VIs are susceptible to interference from environmental factors such as soil noise and atmospheric conditions [30]. In contrast, texture characterizes canopy structure and enhances the sensitivity of remote sensing data to crop physical properties. Consequently, it is less susceptible to external noise and soil background effects, thereby providing more accurate geometric information about land features [35]. Furthermore, texture varies independently of color and brightness, effectively suppressing the occurrence of same-spectrum different-object and same-object different-spectrum phenomena [36]. During the mid-to-late growth stages of alfalfa, canopy structure becomes increasingly complex. At this point, relying solely on spectral information struggles to accurately reflect plant nitrogen status. Texture features, however, effectively capture subtle tonal and structural variations within the canopy, thereby compensating for spectral limitations and significantly improving model estimation accuracy [37]. This study selected the texture indices most correlated with each growth period (NDTI, RTI, and DTI). The components of these indices—mean, hom, sm, corr, and ent—serve to eliminate interference from cluttered overlapping backgrounds, thereby smoothing and homogenizing the images [38]. By filtering key spectral bands such as nir and red-edge, and constructing texture indices based on the texture features within these bands, the canopy structure of alfalfa across its growth stages can be more accurately reflected. This approach reduces overlapping image backgrounds, amplifies spectral differences between objects, and compensates for the insensitivity of vegetation indices to regional size and orientation [39]. Yun et al. [40] demonstrated that texture indices provide complementary information to spectral data, reducing the influence of spectral saturation and environmental factors, thereby enhancing the accuracy of plant PNC estimation. The optimal estimation model achieved an *R*^2^ value of 0.90, consistent with the findings of this study. This indicates that models incorporating both VIs and TIs as input variables can better estimate the nitrogen nutrition status of alfalfa.

### 3.3. Characteristics and Adaptation Differences in Different PNC Prediction Models

In constructing alfalfa PNC estimation models, during the same growth stage and with identical feature combinations, XG-Boost models demonstrated higher estimation accuracy and stability compared to RFR, SVR, and BPNN models. This result stems not only from the inherent advantages of the algorithm itself, but also from the data structure of the feature set used in this study. Under single-feature conditions, the input variable dimension is relatively low, and nonlinear relationships between features are relatively clear. For each growth stage, using VIs or TIs as input variables, XG-Boost achieved average test set *R*^2^ values of 0.70 and 0.66, significantly outperforming RFR (0.60, 0.59), SVR (0.58, 0.56), and BPNN (0.52, 0.54). Furthermore, XG-Boost exhibited a training–validation *R*^2^ difference of less than 0.02 and an *R*^2^ standard deviation of less than 0.02, demonstrating superior overfitting control and prediction stability compared to the other models. The XG-Boost model can leverage its gradient boosting mechanism to keenly capture data feature correlations and precisely identify patterns, enabling relatively accurate PNC estimation [41]. RFR exhibits suboptimal estimation accuracy due to the homogeneity of its learning models and its inability to adequately capture complex biological associations [42]; SVR is sensitive to kernel function selection, and its model generalization capability is limited when handling high-noise or nonlinear complex data [43]; BPNN is sensitive to parameter settings; and neural networks are prone to local optima, resulting in poor fitting performance and significant inversion errors [44]. Under feature fusion conditions, VIs and TIs respectively characterize the spectral response and spatial heterogeneity of the canopy. When fused, they increase the input variable dimension, forming a feature set with strong nonlinearity, interactivity, and a certain degree of redundancy. When using VIs + TIs as input variables for the three growth stages, XG-Boost achieved an average test set *R*^2^ of 0.76, significantly outperforming RFR (0.65), SVR (0.60), and BPNN (0.58). Additionally, XG-Boost exhibited a training–validation *R*^2^ difference below 0.02 and an *R*^2^ standard deviation under 0.015, demonstrating significantly superior overfitting control and prediction stability compared to the other models. Leveraging its gradient boosting mechanism and regularization techniques, the XG-Boost model can effectively identify valid data while avoiding overreliance on noisy data, demonstrating exceptional nonlinear fitting capabilities [45]. Liu et al. [46] found that the XG-Boost model, through gradient boosting and automatic feature selection, prevents overfitting and enables iterative optimization, demonstrating outstanding performance in estimating nitrogen concentration in soybean leaves. Using a fusion of VIs + TIs as input variables, the *R*^2^ value reached 0.82, consistent with the results of this study. The random forest regression (RFR) model, through ensemble decision trees and random feature selection, exhibits good stability and noise resistance. However, its prediction accuracy is lower than XG-Boost but superior to SVR and BPNN models. The Support Vector Regression (SVR) model performed poorly in the high-dimensional features of this study, showing generally low prediction accuracy with insignificant performance improvement after feature fusion. This finding is inconsistent with the results reported by Zhang et al. [21], who achieved an optimal estimation *R*^2^ of 0.87 using an SVR model in their study of nitrogen content estimation based on drone-derived spectral-texture fusion. SVR demonstrated favorable performance after integrating spectral and texture features, effectively enhancing the estimation accuracy of nitrogen content in rice leaves. This discrepancy may stem from inherent differences in input feature characteristics. According to research by Jahaninasab et al. [47], when feature dimensions are high and contain redundant information, kernel-based SVR models are susceptible to the “curse of dimensionality,” leading to degraded generalization performance. Although BP neural networks (BPNNs) possess strong nonlinear mapping capabilities, they are prone to local optima, sensitive to parameters, and exhibit significant prediction fluctuations. Moreover, due to their sensitivity to parameter initialization and network architecture, performance improvements after feature fusion are not significant [44]. In this study, all models achieved training times in the order of seconds, with single-sample prediction latency below 0.01 s. The overall efficiency meets the real-time requirements for drone remote sensing inversion. Among them, XG-Boost and RFR, leveraging the parallel computing capabilities of tree structures, demonstrated superior training efficiency compared to SVR and BPNN while maintaining high accuracy. SVR’s quadratic programming solution mechanism faces scalability bottlenecks in large-sample scenarios. BPNN exhibited the slowest training speed and high sensitivity to parameters. Considering both accuracy and efficiency, XG-Boost emerged as the optimal choice.

### 3.4. Limitations and Prospects

This study achieved satisfactory results in estimating alfalfa plant nitrogen content by integrating spectral and textural information using drone multispectral imagery, though certain limitations remain. Data collection for this study focused on three key growth stages of alfalfa within a single year, and the experimental area was relatively limited. Therefore, the generalizability of the conclusions across different years, varieties, or diverse environmental conditions requires further validation. Furthermore, the limited number of multispectral bands, the absence of multi-source data such as hyperspectral imagery, and the failure to account for environmental factors like soil and moisture restrict the model’s interpretability and adaptability. To address these limitations, subsequent studies should expand to multi-year, multi-varietal, and multi-regional experimental designs to validate the model’s generalizability. Concurrently, integrating multi-source remote sensing data such as hyperspectral and thermal infrared imagery, along with environmental variables like soil nutrients and moisture, can establish a multi-modal fusion model for estimating nitrogen content in alfalfa plants. Despite the aforementioned limitations, the XG-Boost model developed in this study—which integrates vegetation indices and texture indices and achieved an *R*^2^ value of 0.80 on the budding stage validation set—has demonstrated practical application value. It provides rapid, non-destructive nitrogen diagnosis technology support for precision fertilization and smart field management of alfalfa. Additionally, this method can complete single-plot processing within 2–3 h, enabling near-real-time monitoring capabilities. It requires no dedicated hardware development and can directly integrate with existing variable-rate fertilization systems, offering strong commercial integration prospects in large-scale alfalfa cultivation areas.

## 4. Materials and Methods

### 4.1. Study Area and Experimental Design

**Figure 10:**
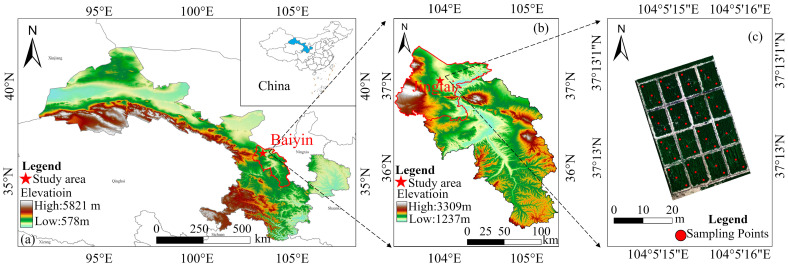
(**a**,**b**) The study area is situated at the Irrigation Experiment Station of the Jingtaichuan Power-Lifted Irrigation Administration Bureau in Baiyin City, Gansu Province, China. (**c**) Distribution of experimental plots and sampling points.

**Figure 11:**
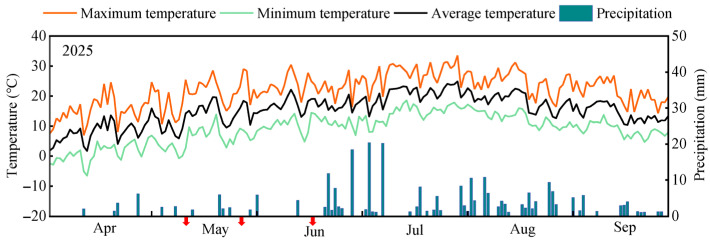
Average daily temperature and average daily precipitation during the alfalfa growing season in 2025. In the figure, the red arrows represent the drone flight dates.

This experiment was conducted from April to October 2025 at the Jingtaichuan Electric Power Lift Irrigation Water Resources Utilization Center Irrigation Experiment Station in Gansu Province (37°12′59″ N, 104°05′10″ E, average elevation 1572 m, Figure 10). The primary soil type at the station is sandy loam, with physicochemical properties (Table 3). The region exhibits a temperate continental arid climate characterized by abundant sunlight and scarce precipitation. Annual average sunshine duration is 2652 h, with a frost-free period of 191 days. Radiation intensity reaches 6.18 × 10^5^ J·cm^−2^, the average temperature is 8.6 °C, annual precipitation is 201.6 mm, and evaporation amounts to 2761 mm. Meteorological data (Figure 11) were monitored by a compact smart agricultural weather station (Davis) installed at the experimental station. The region’s climatic characteristics, including high temperatures, strong evaporation, and low precipitation, influence the migration and transformation processes of nitrogen in the field. While elevated temperatures promote nitrogen metabolism in alfalfa plants, they also exacerbate nitrogen volatilization losses. Concurrently, water stress inhibits soil nitrogen mineralization, further limiting nitrogen availability. Together, these factors constitute the environmental context for remote sensing estimation of plant nitrogen content (PNC) in alfalfa within this study.

### 4.2. Experimental Design

The alfalfa variety tested was Gannong No. 3. Four nitrogen application levels were established: N0 (0), N1 (80 kg·hm^−2^), N2 (160 kg·hm^−2^), and N3 (240 kg·hm^−2^). Potassium and phosphorus fertilizers were applied at local standard rates of 95 kg·hm^−2^ and 850 kg·hm^−2^, respectively. Urea (N ≥ 46%) was used as the nitrogen fertilizer, superphosphate (P_2_O_5_ ≥ 12%) as the phosphorus fertilizer, and potassium sulfate (K_2_O ≥ 50%) as the potassium fertilizer. The fertilizer was dissolved in water, and integrated water and fertilizer drip irrigation technology was used. Potassium and phosphorus fertilizers were applied as basal fertilizers during the regreening stage of the first alfalfa crop. Nitrogen fertilizer was applied in batches during the regreening stage of each alfalfa crop at a ratio of 5:3:2. Each plot measured 54 m^2^ (6 m × 9 m), with 4 replicates, totaling 16 experimental plots (Figure 10c). A completely randomized block design was employed, dividing the 16 plots into 4 blocks (i.e., 4 replications). Within each block, nitrogen levels (N0–N3) were randomly distributed to control for spatial heterogeneity in the field. Additionally, 1 m-wide isolation rows were established between plots, and a 1 m buffer zone was arranged around the perimeter of the experimental field. An independent drip irrigation system was integrated to minimize edge effects and irrigation interference as much as possible. Throughout the trial period, field management practices in each plot were consistent with local standards.

### 4.3. Plant Nitrogen Content Estimation Process

**Figure 12:**
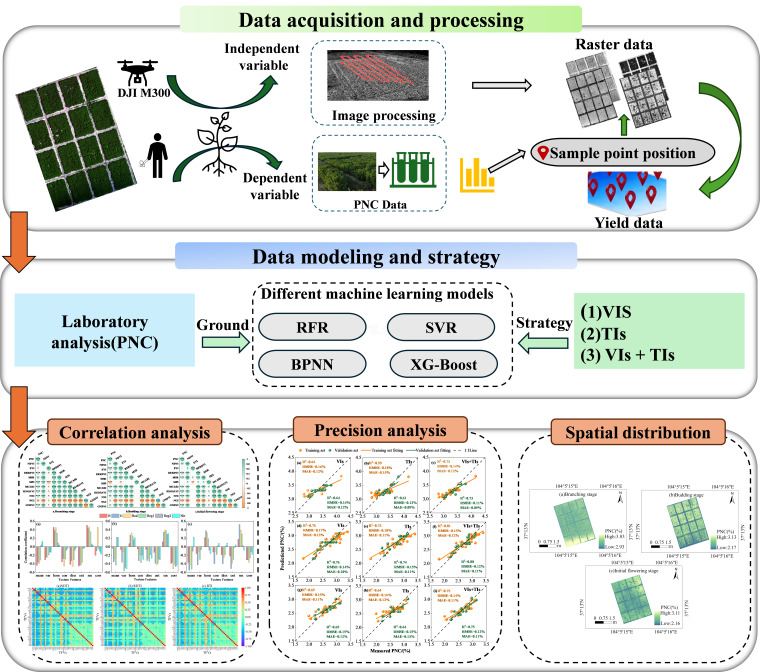
Flowchart for estimating nitrogen content in alfalfa plants.

This study acquired multispectral imagery of alfalfa during three critical growth stages using a multispectral sensor mounted on an unmanned aerial vehicle (UAV). After preprocessing the imagery, vegetation indices, texture features, and texture indices were extracted. Subsequently, Pearson correlation analysis was employed to select the optimal vegetation indices, texture indices, and their combined features. Four machine learning methods—RFR, SVR, BPNN, and XG-Boost—were then applied to construct models for estimating nitrogen content in alfalfa plants (Figure 12).

### 4.4. Data Acquisition and Processing

#### 4.4.1. Plant Nitrogen Content (PNC) Acquisition

While drones acquired multispectral remote sensing imagery, manual collection of alfalfa samples was simultaneously conducted on the ground across 16 plots. Sampling points were uniformly distributed along the diagonals of each plot across the entire field (Figure 10c), employing a five-point sampling method with a 1 m × 1 m sampling frame. After bringing the plants back to the laboratory, we thoroughly washed them. Next, we separated the stems from the leaves and weighed them individually to determine their fresh weight. We placed the samples in bags and transferred them to an oven. We heated the samples at 105 °C for 30 min to kill the tissues, then reduced the temperature to 75 °C to dry them until a constant weight was achieved.

Plant nitrogen concentration (PNC) refers to the total nitrogen content of the stem and leaf composite sample. After drying the alfalfa samples to constant weight, we separately weighed the stem dry mass (SDM) and leaf dry mass (LDM). We grinded the samples using a pulverizer, placed them in resealable bags, and stored them sealed under low-humidity conditions. Leaf nitrogen content (LNC) and stem nitrogen content (SNC) were determined using the Kjeldahl method. Plant nitrogen content was calculated based on the dried sample mass, and alfalfa PNC (%) data were derived using Equation (1).

(1) $$\mathrm{PNC}=\frac{\mathrm{LNC}\;\times \;\mathrm{LDM}+\mathrm{SNC}\;\times \;\mathrm{SDM}}{\mathrm{LDM}+\mathrm{SDM}}$$

#### 4.4.2. Acquisition of Remote Sensing Images

**Figure 13:**
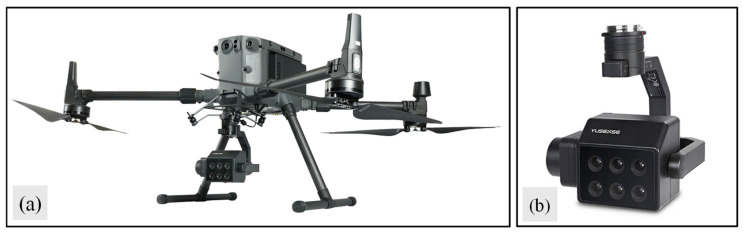
(**a**) DJI Matrice 300 RTK quadcopter drone; (**b**) the MS 600 Pro multispectral camera.

All remote sensing data collection in this experiment was conducted under clear, windless conditions with ample sunlight. Data acquisition occurred during the branching stage (10 May 2025), budding stage (24 May 2025), and initial flowering stage (15 June 2025); a DJI Matrice 300 RTK quadcopter drone (DJI Technology Co., Ltd., Shenzhen, China) (Figure 13a) equipped with an MS 600 Pro multispectral camera (Changguang Yuchen Information Technology Equipment Co., Ltd., Qingdao, China) (Figure 13b) was utilized. Prior to data collection, radiometric calibration was performed using a standardized white reference panel to compensate for variations in lighting conditions. Each flight followed a predetermined route, with the drone operating at a speed of 2.7 m/s at an altitude of 30 m. The heading overlap rate was 80%, the side overlap rate was 70%, and the flight duration was from 12:00 to 13:00. The multispectral camera was oriented vertically downward. The center wavelengths, bandwidths, and reflectance of the diffuse reflector (Table 4) yielded a ground resolution of 2.16 cm.

#### 4.4.3. Remote Sensing Image Preprocessing

In this study, four ground control points (GCPs) were set up within the study area, and their coordinates were accurately measured using real-time kinematic (RTK) positioning technology. Image stitching and georeferencing were performed using Pix4D Mapper 4.8.0 software, with manual ground control point (GCP) marking to enhance positioning accuracy. The resulting digital orthophoto map exhibited root mean square errors (RMSEs) of 0.22 m, 0.31 m, and 0.12 m along the x, y, and z axes, respectively, indicating high spatial accuracy. Radiometric calibration was then carried out using a standardized white reference panel to guarantee the accuracy of the spectral data for the canopy. Following geometric and radiometric calibration, ENVI 5.6 software converted the Digital Number (D_N_) values of the calibrated imagery into reflectance. We calculated the mean reflectance spectrum of the alfalfa canopy within the region of interest (ROI) to represent the spectral reflectance for that sampling point, and extracted reflectance data across different spectral bands.

### 4.5. Feature Extraction

#### 4.5.1. Calculation of Vegetation Indices

Based on data acquired by the multispectral camera, the central bands include the red band (R), blue band (B), green band (G), near-infrared (NIR) band, red-edge 1 band, and red-edge 2 band. Ten VIs were selected for this study (Table 5). Using ENVI 5.6 software, VI images were generated by combining reflectance images from different bands. ArcGIS Pro 3.1.6 software was employed to create vector maps for each experimental plot and assign attribute codes. Using ArcGIS’s Zonal Statistics tool to process vector maps and VI images of the study area, the average VI value within the area of interest for each experimental plot was calculated. 

#### 4.5.2. Texture Feature Extraction

The texture information addressed in this paper includes texture feature values (TFVs) and texture indices (TIs). Using the gray-level co-occurrence matrix (GLCM) method in ENVI 5.6 with a sliding window size of 9 × 9, this dimension balances canopy structural detail with computational efficiency and aligns with the recommended window size in prior studies on rice nitrogen content [31]. The pixel spacing was set to 1 to capture spatial dependencies between adjacent pixels; excessive spacing would reduce sensitivity to fine canopy textures. The grayscale level was set to 64 to preserve texture information while effectively controlling feature dimensionality. Texture directions were averaged across 0°, 45°, 90°, and 135° to eliminate anisotropic interference caused by canopy row and spacing orientations. Eight TFVs were obtained: mean (mean), variance (var), homogeneity (hom), contrast (con), dissimilarity (diss), entropy (ent), correlation (corr), and second moment (sm) [55]. The MS 600 Pro multispectral camera features 6 spectral bands, yielding a total of 48 TFVs across all growth stages. In this study, TFVs are named as “band + texture feature values” (e.g., mean value of the red band (R) is denoted as R_mean). For each band, texture feature images were processed by defining regions of interest (ROIs). The texture values within these defined regions were then extracted and used as the texture feature values for the respective areas.

Three texture indices (TIs) constructed from TFV combinations are defined as follows: Normalized Difference Texture Index (NDTI), Ratio Texture Index (RTI), and Renormalized Difference Texture Index (RDTI). Their calculation formulas are as follows:

(2) $$\mathrm{NDTI}\;=\;\left(T_{1}\;-\;T_{2}\right)/\left(T_{1}\;+\;T_{2}\right)$$

(3) $$\mathrm{RTI}=T_{1}/T_{2}$$

(4) $$\mathrm{RDTI}=\left(T_{1}\;-\;T_{2}\right)/\sqrt{\left(T_{1}\;+\;T_{2}\right)}$$

In the formulas, T1 and T2 represent any two TFVs selected from the 48 TFVs extracted from the spectral bands. Therefore, each category of TIs can form 2304 distinct combinations of texture indices.

### 4.6. Pearson Correlation Analysis

The multispectral remote sensing images studied comprise six bands, with eight texture features extracted from each band. Texture indices between two texture features were constructed, and ten vegetation indices were calculated. Directly utilizing all feature variables would lead to data redundancy and increased computational complexity. The Pearson correlation coefficient method is a statistical technique used to assess the strength and direction of a linear relationship between two variables. It effectively eliminates redundant information during variable selection, making it widely applied in practical research [56]. Its values range from −1 to 1. A higher absolute value of r indicates stronger linear correlation between the predictor and target variables. Based on the Pearson correlation coefficient evaluation criteria, |r| ≥ 0.8 signifies a high correlation; 0.5 ≤ |r| < 0.8 denotes a moderate correlation; 0.2 ≤ |r| < 0.5 indicates a low correlation; and |r| < 0.2 suggests negligible correlation. Correlation analysis was performed for vegetation indices, texture features, and texture indices using the following calculation formula:

(5) $$r\;=\;\frac{\sum \left(X_{i}-\overset{-}{X}\right)\left(Y_{i}-\overset{-}{Y}\right)}{\sqrt{\sum \left(X_{i}-\overset{-}{X}\right)\cdot }\sqrt{\sum \left(Y_{i}-\overset{-}{Y}\right)}}$$

In the equation, r represents the Pearson correlation coefficient, $X_{i}$ and $Y_{i}$ denote the observed values of two variables, and $\overset{-}{X}$ and $\overset{-}{Y}$ denote the sample means of the variables.

### 4.7. Model Construction and Accuracy Evaluation

This study selected four models—RFR, SVR, BPNN, and XG-Boost—to ensure both diversity in model types and consideration of their established application backgrounds in remote sensing-based agricultural parameter inversion. This approach enables a systematic and comprehensive evaluation of the applicability of different machine learning methods for estimating alfalfa PNC, providing a comparable benchmark for subsequent research. The model input variables were divided into three groups: the vegetation index variable group, the texture index variable group, and the vegetation index + texture index variable group. Four machine learning models—random forest regression (RFR), Support Vector Regression (SVR), Backpropagation Neural Network (BPNN), and XG-Boost—were employed to establish alfalfa plant nitrogen (PNC) estimation models for different input variable groups across various growth stages. As an ensemble learning algorithm, RFR builds several decision trees through the Bootstrap sampling technique and random feature selection. The predictions from these trees are averaged to produce the output of the random forest regression, enhancing the model’s diversity and robustness [57]. SVR is a method that applies Support Vector Machine (SVM) to address regression problems. It constructs an optimized model by minimizing empirical loss and maximizing the margin between the decision boundaries, thereby enhancing the model’s generalization ability [58]. BPNN is among the most commonly used algorithms in artificial neural networks, possessing strong nonlinear fitting capabilities. It iteratively refines weight parameters through the backpropagation algorithm to minimize the discrepancy between predicted and actual values [59]. XG-Boost is a machine learning technique that operates within a gradient boosting framework. This algorithm simultaneously incorporates model performance enhancement and complexity control into its optimization objective, enabling efficient handling of large-scale datasets and complex problems [45].

Hyperparameter optimization for the model employs grid search and 4-fold cross-validation to ensure optimal model performance. In random forest regression (RFR), model training utilizes the greedy splitting strategy of the CART algorithm, with the number of decision trees set to 5 and maximum depth to 3. The model trains directly to the preset number of decision trees. Support Vector Regression (SVR) model optimization was achieved through the Sequential Minimal Optimization (SMO) algorithm, which decomposes the original quadratic programming problem into a series of minimal subproblems for iterative solutions. The model was iterated on the training set until the KKT conditions were satisfied. A radial basis function kernel was employed with penalty parameters C = 100 and ε = 0.1, and both input features and target variables underwent standardization processing. The BP neural network (BPNN) employs a two-layer hidden structure with the ‘relu’ activation function. The optimizer uses ‘adam’, with an initial learning rate of 0.001 and adaptive learning rate adjustment enabled. To prevent overfitting, L2 regularization (α = 0.001) was applied to control model complexity. Model training employed sklearn’s default early stopping strategy: training automatically terminates when validation loss improvement falls below the tolerance threshold (tol = 1 × 10^−4^) for 10 consecutive iterations. Similar to SVR, BPNN inputs features and target variables undergo standardized preprocessing. For the XG-Boost model, the number of boosting trees is set to 5, maximum tree depth to 2, and learning rate to 0.4. Model optimization employed gradient boosting, approximating the loss function via second-order Taylor expansion and constructing regression trees using a greedy algorithm. The objective function incorporated both L1 and L2 regularization terms to constrain model complexity, training directly to the preset number of boosting trees. As tree models, RFR and XG-Boost are insensitive to feature scale, allowing direct use of raw data inputs without normalization. In this study, 48 datasets were collected for each growth stage of alfalfa. Two-thirds of the samples were randomly selected as the training set, while the remaining one-third served as the validation set. This random division was repeated ten times to eliminate single-division randomness. The final accuracy metric was calculated as the mean of ten results (with the standard deviation of the validation set *R*^2^ consistently below 0.02), indicating that model evaluation was minimally affected by random division and yielded robust results. To evaluate model performance, this study selected the coefficient of determination (*R*^2^), root mean square error (RMSE), and mean absolute error (MAE) as evaluation metrics for both the model training and validation phases. Generally, the closer *R*^2^ is to 1, and the lower the RMSE and MAE values, the stronger the model’s stability and the more concentrated its prediction results [60,61].

### 4.8. Data Processing

Data organization in this study was carried out using Microsoft Excel 2019. Preprocessing of drone multispectral imagery was conducted using Pix4D Mapper 4.8.0 and ENVI 5.6 software to extract canopy mean reflectance and TFVs, subsequently calculating vegetation indices (VIs) and texture indices (TIs). Using ArcGIS Pro 3.1.6, we extracted datasets for each sample point based on the vector boundaries of the experimental plots. We performed Pearson correlation analysis using Python 2024.3.5 programs and built and tested models based on the sklearn library. Plots were generated using Origin 2021 software.

## 5. Conclusions

This study made use of multispectral UAV imagery to build and compare various machine learning models by integrating vegetation and texture indices. The key findings are as follows:

(1) During the three growth stages of alfalfa, the correlation coefficients |r| between vegetation indices such as SIPI, MCARI, REOSAVI, EVI, RERDVI, NNI, and GNDVI and PNC ranged from 0.56 to 0.68 (*p* < 0.001). Compared to single texture features, texture indices constructed through combination significantly enhanced correlation with PNC. The selected NDTI, RTI, and RDTI all exhibited |r| values above 0.6 (*p* < 0.001), effectively supplementing vegetation indices.
(2) Combining vegetation and texture indices notably enhances the accuracy of alfalfa PNC estimation models. Compared to single features, the integrated features increased the *R*^2^ values at the branching stage, budding stage, and initial flowering stage by 5.4% to 19.7%, 1.7% to 16.4%, and 5.2% to 17.2%, respectively.
(3) Under different growth stages and input variables, the XG-Boost model consistently demonstrated optimal performance, achieving the highest estimation accuracy when using VIs + TIs as input variables. Specifically, the validation set *R*^2^ for the branching stage was 0.73 with an RMSE of 0.11%; the validation set during the budding stage yielded an *R*^2^ of 0.80 and an RMSE of 0.12%; and the validation set during the initial flowering stage achieved an *R*^2^ of 0.75 and an RMSE of 0.12%.

To conclude, combining multispectral vegetation and texture indices from UAV imagery with an XG-Boost model allows for precise and efficient monitoring of nitrogen content in alfalfa plants. This method offers a theoretical foundation for precision fertilization and the intelligent management of alfalfa crops.

## Figures and Tables

**Table 1:** Statistics on PNC characteristics at each fertility stage.

Category	Observations	Min/%	Max/%	Mean/%	SD/%	CV/%
Branching stage	48	2.82	4.16	3.31	0.26	7.90
Budding stage	48	1.80	3.39	2.75	0.33	12.04
Initial flowering stage	48	1.99	3.14	2.64	0.27	10.23
All datasets	144	1.80	4.16	2.90	0.41	14.03

**Table 2:** Prediction accuracy of the PNC validation set based on different machine learning models.

Stages	Feature	Metrics	RFR	SVR	BPNN	XG-Boost
Branching stage	VIs	*R* ^2^	0.58	0.56	0.53	0.64
		RMSE (%)	0.20	0.16	0.20	0.14
		MAE (%)	0.13	0.11	0.15	0.12
	TIs	*R* ^2^	0.58	0.54	0.52	0.61
		RMSE (%)	0.20	0.13	0.21	0.12
		MAE (%)	0.15	0.11	0.18	0.09
	VIs + TIs	*R* ^2^	0.62	0.59	0.59	0.73
		RMSE (%)	0.15	0.19	0.18	0.11
		MAE (%)	0.11	0.11	0.14	0.09
Budding stage	VIs	*R* ^2^	0.64	0.59	0.52	0.76
		RMSE (%)	0.24	0.22	0.26	0.14
		MAE (%)	0.20	0.19	0.23	0.10
	TIs	*R* ^2^	0.61	0.56	0.54	0.74
		RMSE (%)	0.23	0.23	0.25	0.15
		MAE (%)	0.19	0.20	0.21	0.11
	VIs + TIs	*R* ^2^	0.71	0.60	0.58	0.80
		RMSE (%)	0.21	0.20	0.24	0.12
		MAE (%)	0.18	0.19	0.20	0.11
Initial flowering stage	VIs	*R* ^2^	0.58	0.58	0.51	0.69
		RMSE (%)	0.17	0.15	0.17	0.15
		MAE (%)	0.13	0.12	0.12	0.12
	TIs	*R* ^2^	0.59	0.57	0.56	0.64
		RMSE (%)	0.15	0.21	0.11	0.15
		MAE (%)	0.11	0.18	0.09	0.13
	VIs + TIs	*R* ^2^	0.63	0.61	0.58	0.75
		RMSE (%)	0.19	0.15	0.19	0.12
		MAE (%)	0.15	0.13	0.16	0.11

**Table 3:** Soil physicochemical properties.

Index	Numeric Value	Unit
Dry bulk density	1.35	g·cm^−3^
Field capacity	24.6%	—
pH	8.10	—
Organic matter	6.07	g·kg^−1^
Total nitrogen	1.68	g·kg^−1^
Total phosphorus	1.37	g·kg^−1^
Total potassium	34.09	g·kg^−1^
Fast-acting nitrogen	74.49	mg·kg^−1^
Fast-acting phosphorus	33.15	mg·kg^−1^
Fast-acting potassium	148.39	mg·kg^−1^

**Table 4:** The central wavelength and reflectivity of the diffuse reflector.

Spectral Band	Center Wavelength/nm	Bandwidth/nm	Reflectance of Diffuse Reflector/%
Blue	450	35	60
Green	555	25	60
Red	660	20	60
Red-edge 1	720	10	60
Red-edge 2	750	15	60
NIR	840	35	60

**Table 5:** Vegetation indices and calculation formulas used in this paper.

Vegetation Index (VI)	Formula	Reference
Normalized difference vegetation index (NDVI)	(NIR−R)/(NIR+R)	[48]
Enhanced vegetation index (EVI)	2.5(NIR−R)/(NIR+6R−7.5B+1)	[49]
Red-edge re-normalized difference vegetation index (RERDVI)	$(\mathrm{NIR}-RE1)/{\left(NIR+RE1\right)}^{0.5}$	[50]
Modified simple ratio (MSR)	$(\mathrm{NIR}/R-1)/{(\mathrm{NIR}/R+\;1)}^{0.5}$	[51]
Structure-insensitive pigment index (SIPI)	(NIR−B)/(NIR−R)	[51]
Modified chlorophyll absorption in reflectance index (MCARI)	[(NIR−RE1)−0.2(NIR−R)](NIR/RE1)	[52]
Red-edge optimized soil-adjusted vegetation index (REOSAVI)	(1+0.16)(NIR−RE1)/(NIR+RE1+0.16)	[50]
Normalized near-infrared index (NNI)	NIR/(NIR+RE1+G)	[53]
Normalized greenness index (NGI)	G/(NIR+G+RE1)	[54]
Green normalized difference vegetation index (GNDVI)	(NIR−G)/(NIR+G)	[54]

In the table, B, G, R, RE1, RE2, and NIR represent the spectral reflectance of the MS 600 Pro multispectral camera at wavelengths of 450 nm, 555 nm, 660 nm, 720 nm, 750 nm, and 840 nm, respectively.

## Data Availability

All data are incorporated into the article.
